# Flexible thermoelectric films formed using integrated nanocomposites with single-wall carbon nanotubes and Bi_2_Te_3_ nanoplates via solvothermal synthesis

**DOI:** 10.1038/s41598-020-73808-4

**Published:** 2020-10-12

**Authors:** Hayato Yabuki, Susumu Yonezawa, Rikuo Eguchi, Masayuki Takashiri

**Affiliations:** Department of Materials Science, Tokai University, Hiratsuka, Kanagawa 259-1292 Japan

**Keywords:** Nanoscale materials, Carbon nanotubes and fullerenes, Nanoparticles

## Abstract

Single-wall carbon nanotubes (SWCNTs) and Bi_2_Te_3_ nanoplates are very promising thermoelectric materials for energy harvesting. When these two materials are combined, the resulting nanocomposites exhibit high thermoelectric performance and excellent flexibility. However, simple mixing of these materials is not effective in realizing high performance. Therefore, we fabricated integrated nanocomposites by adding SWCNTs during solvothermal synthesis for the crystallization of Bi_2_Te_3_ nanoplates and prepared flexible integrated nanocomposite films by drop-casting. The integrated nanocomposite films exhibited high electrical conductivity and an n-type Seebeck coefficient owing to the low contact resistance between the nanoplates and SWCNTs. The maximum power factor was 1.38 μW/(cm K^2^), which was 23 times higher than that of a simple nanocomposite film formed by mixing SWCNTs during drop-casting, but excluding solvothermal synthesis. Moreover, the integrated nanocomposite films maintained their thermoelectric properties through 500 bending cycles.

## Introduction

Nanocomposite materials, which are formed by mixing two or more dissimilar materials at the nanoscale, have attracted considerable attention in various industrial fields such as electronics and biotechnology^1–4^, because they have new and improved structures and properties compared to the corresponding materials formed at the macroscale. Carbon nanotubes (CNTs) are one of the most promising components in nanocomposite materials^5–7^. The flexibility, strength, and electrical conductivity of the materials can be increased by introducing CNTs^8,9^. There are generally two types of CNTs: single-walled CNTs (SWCNTs) and multi-walled CNTs (MWCNTs). SWCNTs exhibit semiconducting properties determined by their chirality^10^ and are used in electronic devices, such as field-effect transistors and solar cells^11–13^.

Recently, it was found that SWCNTs exhibit relatively high thermoelectric properties^14–16^. SWCNTs are also inherently flexible; hence, they can be used to create flexible thermoelectric generators^17–20^. In addition, their thermoelectric properties can be improved by forming nanocomposites with organic thermoelectric materials^21–25^. To further improve the thermoelectric properties of the nanocomposite materials while maintaining their flexibility, one promising approach is to merge SWCNTs with nanosized-inorganic thermoelectric materials, such as bismuth telluride-based alloys, which exhibit the best thermoelectric properties at approximately 300 K and an n-type Seebeck coefficient (– 150 to – 200 µV/K)^26–29^. Materials composed of SWCNTs and bismuth telluride-based alloys are available as n-type nanocomposites, even though normal SWCNTs exhibit p-type Seebeck coefficients (50–60 µV/K)^30,31^. Moreover, the structure of bismuth telluride-based alloys is important because the thermoelectric properties depend on the size and dimension of the material. The thermoelectric properties improve as the size and dimension decrease due to the quantum confinement effect and phonon scattering at the boundaries^32,33^.

In our previous study, to reduce the size and dimension of bismuth telluride-based alloys, single-crystalline Bi_2_Te_3_ nanoplates (quasi-2D material) with a thickness of several tens of nanometers were synthesized using a solvothermal method^34,35^. After the nanoplate fabrication, flexible thin films were prepared by mixing the Bi_2_Te_3_ nanoplates and SWCNTs by drop-casting^36,37^. Although the Bi_2_Te_3_ nanoplates and SWCNTs had high electrical conductivities individually, the conductivity decreased after mixing, owing to the high contact resistance between them.

In order to decrease the contact resistance and improve the thermoelectric properties of the nanocomposite materials, it is necessary to integrate the SWCNTs and Bi_2_Te_3_ nanoplates. Liu et al. fabricated an integrated nanocomposite of Bi_2_Te_3_ nanoparticles and SWCNTs by hydrothermal synthesis, and pressed the resulting nanocomposites into bulk samples^38^. Jin et al. developed flexible thermoelectric materials by fabricating a hybrid nanocomposite comprising highly ordered Bi_2_Te_3_ nanocrystals anchored on an SWCNT network using magnetron sputtering^28^. These pioneering studies motivated us to fabricate integrated nanocomposites of SWCNTs and Bi_2_Te_3_ nanoplates using a solvothermal synthesis and to produce flexible thin films with the nanocomposites.

Here, we report the fabrication of integrated nanocomposites with SWCNTs and Bi_2_Te_3_ nanoplates via solvothermal synthesis. Flexible films were formed using the integrated nanocomposites by drop-casting followed by heat treatment. The thermoelectric properties of the films were measured at approximately 300 K and compared to those of the simple nanocomposite thin films that were produced by adding SWCNTs during drop-casting, but excluding solvothermal synthesis.

## Results

### Structural properties of the integrated nanocomposite films

**Figure 1:**
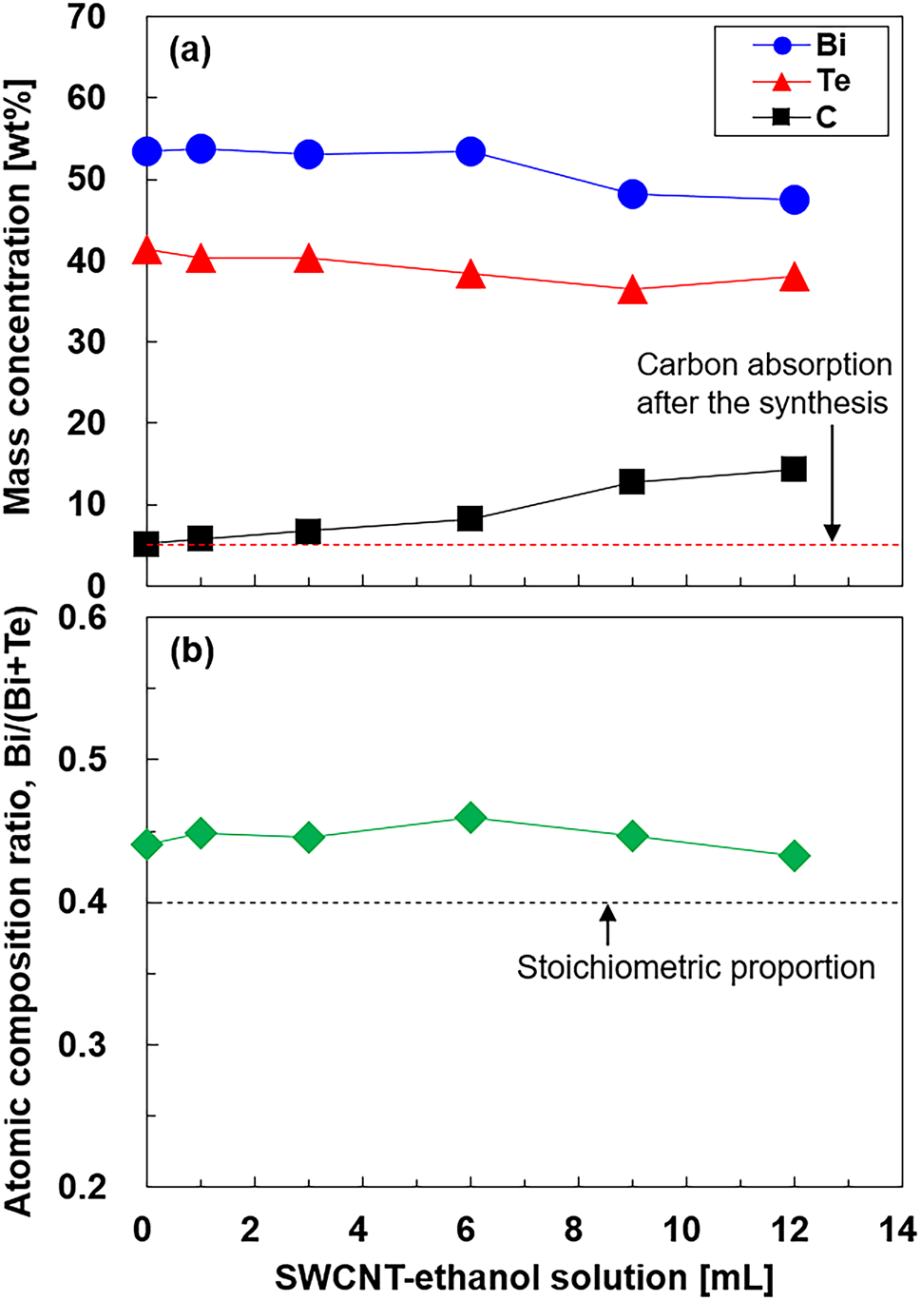
(**a**) Mass concentration of bismuth (Bi), tellurium (Te), and carbon (C); (**b**) bismuth atomic composition ratio [Bi/(Bi + Te)] in nanocomposite films as a function of the SWCNT-ethanol solution measured using EPMA.

Figure 1 presents the component analysis of the integrated nanocomposites determined by electron probe microanalysis (EPMA). The mass concentrations of bismuth, tellurium, and carbon in the samples are shown in Fig. 1a. Carbon (5.2 wt%) was detected in a sample with no SWCNTs because of the adsorbed CO_2_ gas and organic matter found on the nanoplate surfaces. Therefore, the mass concentration of carbon derived from SWCNTs in the integrated nanocomposite films can be obtained by subtracting from that of the sample with no SWCNTs. When the SWCNT-ethanol solution in the precursor solution increased from 1 to 12 mL, the mass concentration of carbon increased from 5.8 to 14.3 wt%. On the contrary, the mass concentrations of bismuth and tellurium decreased as the SWCNT-ethanol solution increased. Therefore, even if SWCNTs were contained in the precursor solution, precipitates composed of bismuth and tellurium atoms were formed during the solvothermal synthesis. The bismuth atomic composition ratio of the samples, [Bi/(Bi + Te)], is shown in Fig. 1b and was calculated to be approximately 0.45 for all SWCNT-ethanol solutions, which was higher than the stoichiometric proportion of 0.40. This phenomenon has been observed in Bi_2_Te_3_ nanoplates with no SWCNTs formed via solvothermal synthesis in our previous studies^39,40^. The mechanism of this phenomenon is due to differences in the production of Bi^3+^ cations and Te^2^^−^ anions, where Bi_2_Te_3_ is formed via the following chemical reaction: Bi_2_Te_3_ ← 2Bi^3+^ + 3Te^2^^−^. Bi^3+^ cations are relatively easy to produce in aqueous solutions, but Te^2^^−^ anions are produced via complicated processes^34^. Therefore, the number of Te^2^^−^ anions tended to be less than that of Bi^3+^ cations.

**Figure 2:**
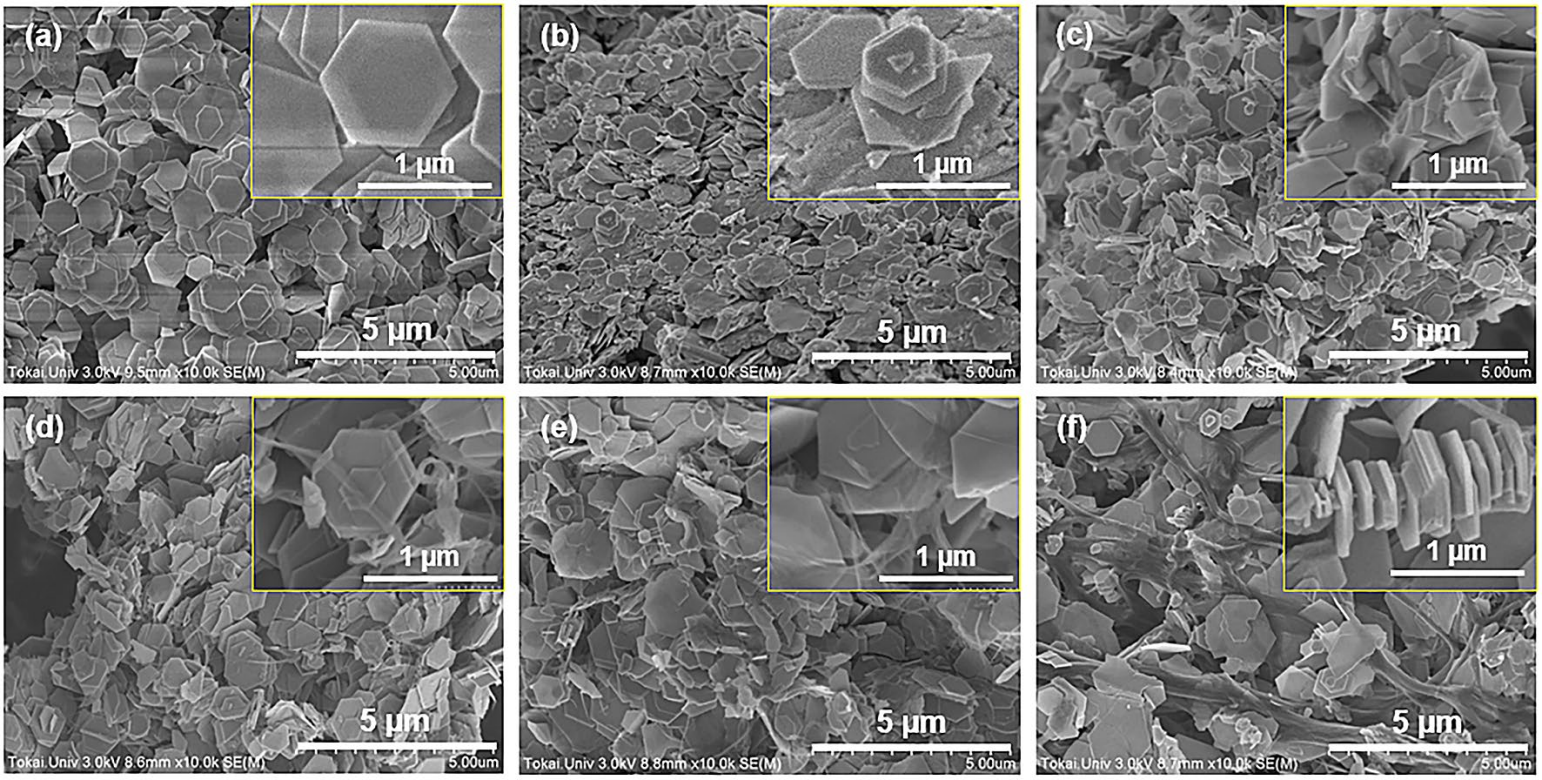
Surface SEM images of the nanocomposite films. (**a**)–(**f**) correspond to SWCNT-ethanol solution of 0, 1, 3, 6, 9, and 12 mL, respectively.

Figure 2 shows the surface images of the integrated nanocomposite films with varying SWCNT-ethanol solutions in the precursor solution, obtained by scanning electron microscopy (SEM). In Fig. 2a, the nanoplate film with no SWCNTs shows regular hexagonal Bi_2_Te_3_ nanoplates that are well aligned with an average diameter of approximately 1 μm. Even though the SWCNT-ethanol solution was increased to 1 mL, as shown in Fig. 2b, the structure of the SWCNTs was not observed in the film. When the SWCNT–ethanol solution was greater than 3 mL, bundles of SWCNTs integrated with the Bi_2_Te_3_ nanoplates were clearly observed (Fig. 2c–e). At a SWCNT-ethanol solution of 12 mL, the diameter of the SWCNT bundles increased and the beaded Bi_2_Te_3_ nanoplates grew perpendicular to the former (Fig. 2f). As the SWCNT-ethanol solution increased, the shape of the nanoplates gradually deviated from the regular hexagon, even though the bismuth atomic composition ratio [Bi/(Bi + Te)] was nearly constant in all samples. Typical images of the nanoplates analyzed by transmission electron microscopy (TEM) are presented in Supplementary Fig. S1. A possible explanation for the change in shape of the Bi_2_Te_3_ nanoplates is that the surface adsorbates derived from the SWCNTs on the Bi_2_Te_3_ nanoplates affected the crystal growth. The images of the nanoplate surfaces analyzed by high-resolution TEM are presented in Supplementary Fig. S2. According to this figure, all nanoplates exhibited lattice fringes, indicating that the nanoplates comprised a single-crystalline phase; however, surface adsorbates were observed in large areas, thus limiting regular crystal growth.

**Figure 3:**
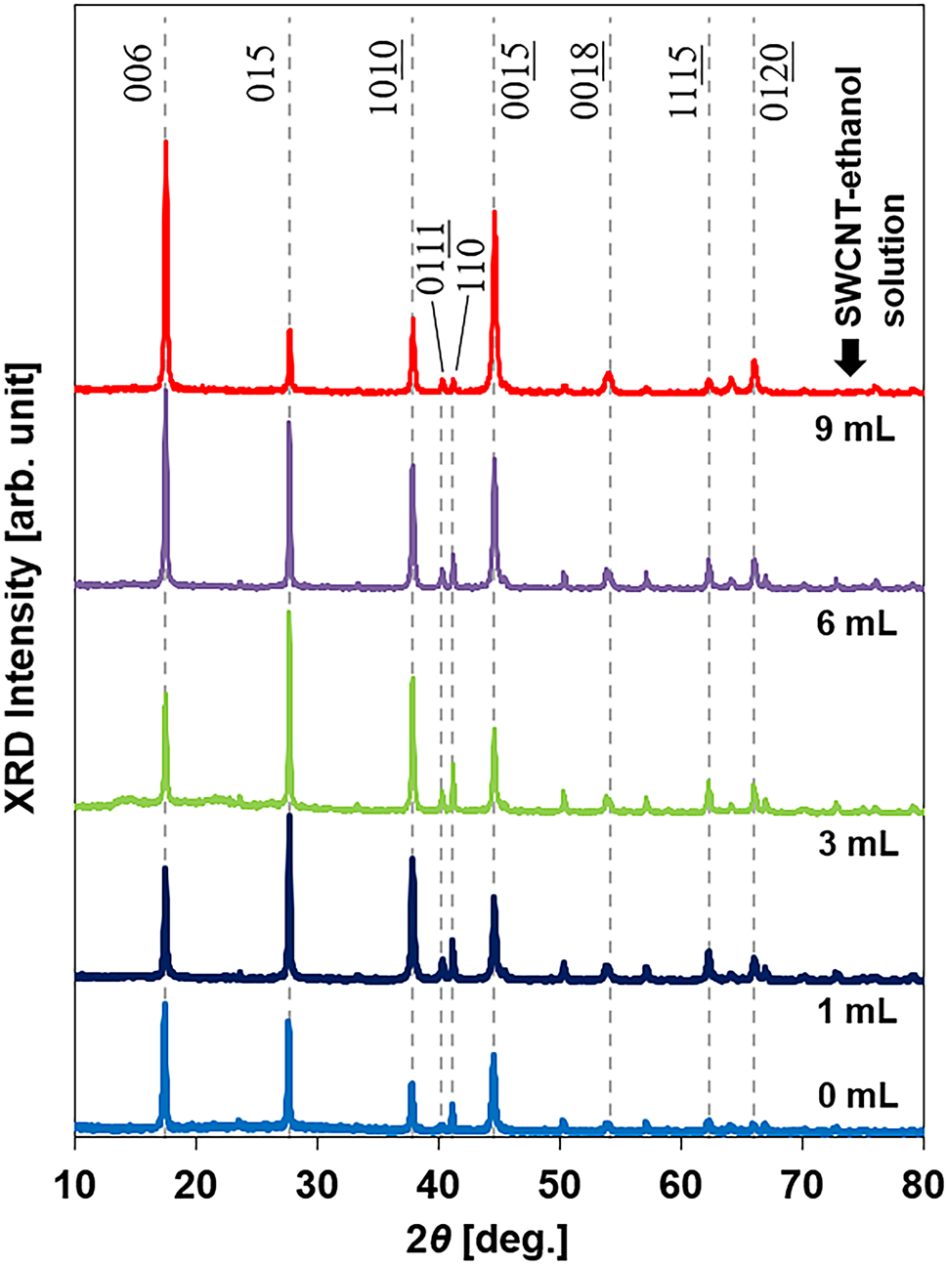
XRD patterns of the nanocomposite films obtained for various SWCNT-ethanol solutions.

**Figure 4:**
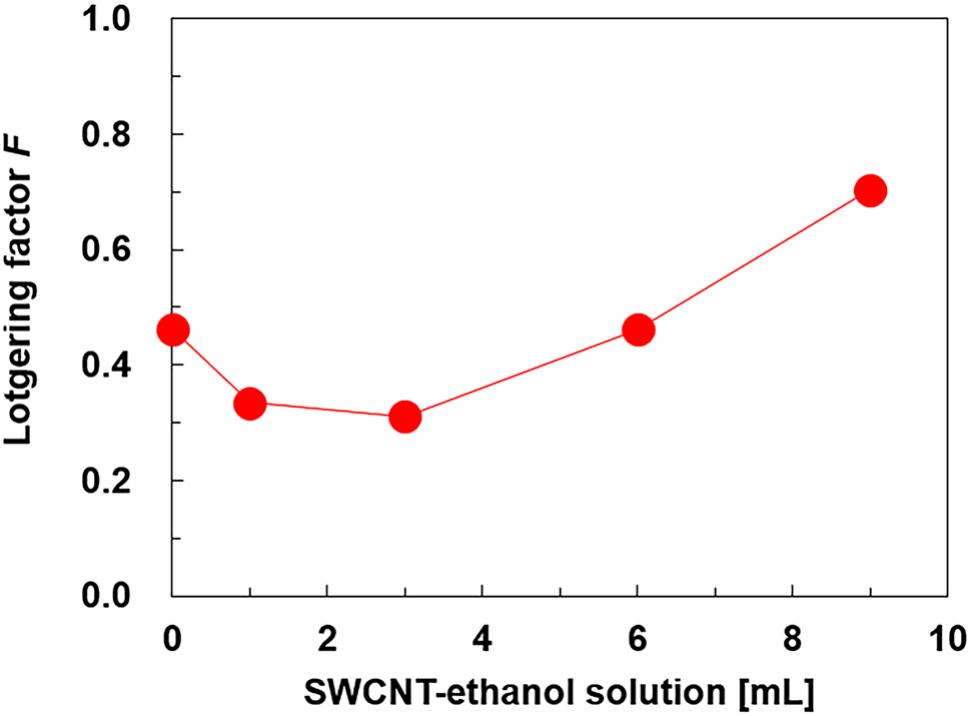
Lotgering factor of the nanocomposite films obtained for various SWCNT-ethanol solutions.

The X-ray diffraction (XRD) patterns of the integrated nanocomposite films with various SWCNT-ethanol solutions in the precursor solution are presented in Fig. 3. The XRD pattern of the film at a SWCNT-ethanol solution of 12 mL was not measured because of the fragile film structure. The XRD patterns for all samples can be indexed to the standard Bi_2_Te_3_ diffraction pattern (JCPDS 15–0863) even though the bismuth atomic composition ratio deviated from the stoichiometric proportion by a maximum of 6%, and the shape of the nanoplates deviated from a regular hexagon. As shown in Fig. 4, we investigated the degree of alignment in the integrated nanoplate films using the Lotgering factor, *F*, which was calculated using Eq. (1)^41–43^:

1 $$F = \frac{{P - P_{0} }}{{1 - P_{0} }},$$

where *P*_0_ = Σ*I*_0_(00* l*)/Σ*I*_0_(*hkl*) and *P* = Σ*I*(00* l*)/Σ*I*(*hkl*). *I*_0_ and *I* denote the peak intensities in the XRD patterns of the standard (JCPDS 15–0863) and of the obtained Bi_2_Te_3_ films, respectively. An *F* value of 1.0 indicates that the basal plane of all nanoplates is parallel to the substrate, whereas an *F* value of zero indicates that the nanoplates randomly stack on the substrate. The film with no SWCNTs exhibited an *F* value of 0.46. When the SWCNT-ethanol solution increased to 3 mL, the *F* value decreased to 0.31. This indicated that the nanoplates were stacked in a relatively disordered condition. Upon further increasing the SWCNT amount, the *F* value of the film increased. At a SWCNT-ethanol solution of 9 mL, the film exhibited an *F* value of 0.70, indicating that a certain amount of SWCNTs facilitated the alignment of the nanoplates. The mechanism that increases the *F* value at an SWCNT-ethanol solution of 9 mL has not yet been clarified. However, a possible explanation is that a certain number of Bi_2_Te_3_ nanoplates in the precursor solution grew perpendicular to the SWCNT surfaces. When the amount of SWCNTs increased in the solution, the number of well-aligned Bi_2_Te_3_ nanoplates on the SWCNT surfaces increased, leading to an increase in the *F* value.

### Thermoelectric properties of integrated nanocomposite films

**Figure 5:**
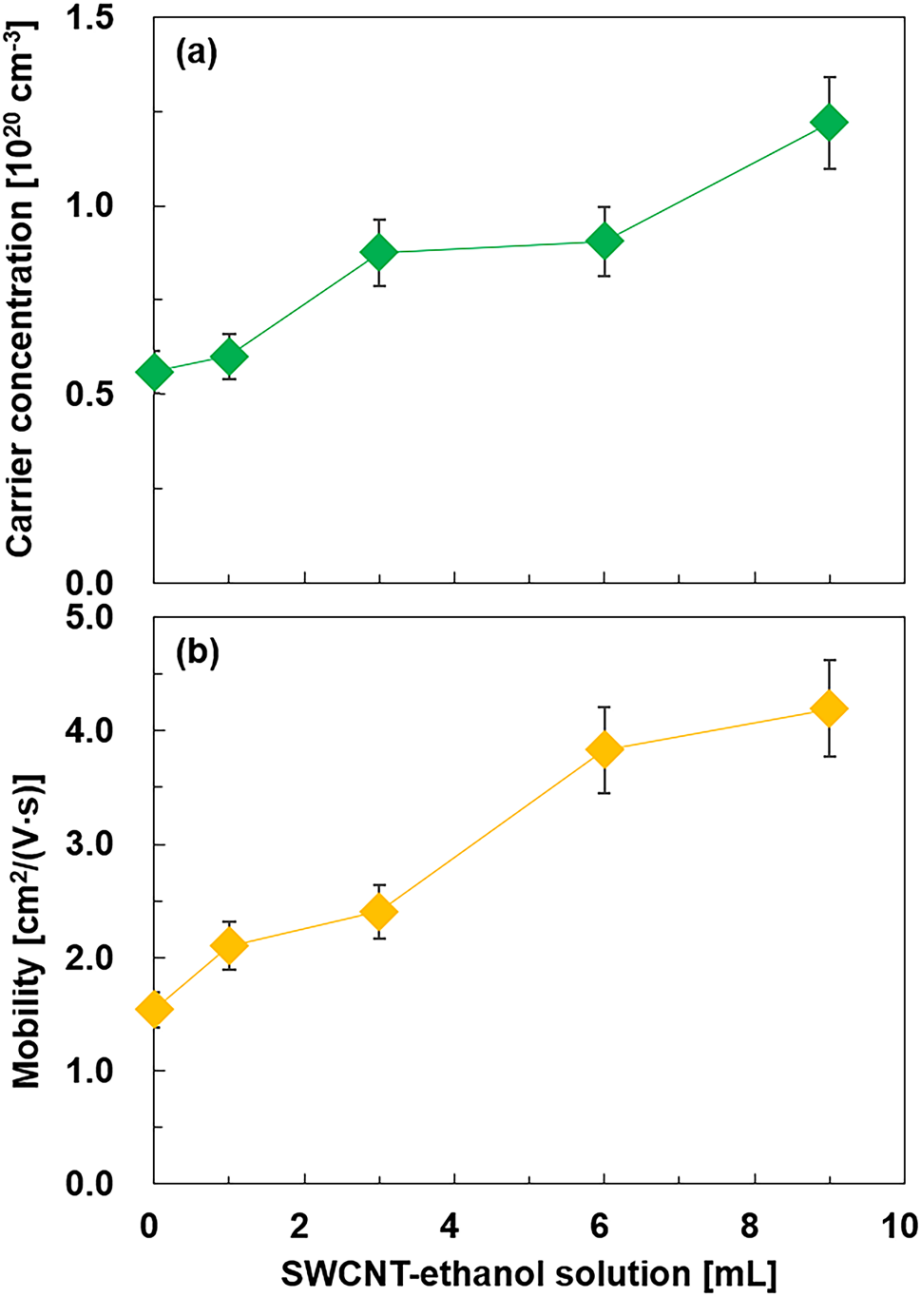
Electronic transport properties of the nanocomposite films as a function of the SWCNT-ethanol solution: (**a**) carrier concentration and (**b**) mobility.

Figure 5 shows the electronic transport properties of the integrated nanocomposite film analyzed using Hall effect measurements. The electronic transport properties of the film in a SWCNT-ethanol solution of 12 mL were not measured because of the fragility of the sample. As shown in Fig. 5a, the carrier concentration of the nanoplate film with no SWCNTs was 5.6 × 10^19^ cm^−3^, which is similar to that of undoped single-crystalline Bi_2_Te_3_ bulk compounds fabricated using a traveling heat method^44^. The carrier concentration increased linearly as the SWCNT-ethanol solution increased. The integrated nanocomposite film with a 9 mL SWCNT-ethanol solution exhibited a carrier concentration of 1.2 × 10^20^ cm^−3^. As shown in Fig. 5b, the mobility of the nanoplate film with no SWCNTs was 1.5 cm^2^/(V s). The mobility increased linearly as the SWCNT-ethanol solution increased, reaching 4.0 cm^2^/(V s) in 9 mL SWCNT-ethanol solution. Therefore, the increase in the amount of SWCNTs in the films contributed to an increase in the carrier concentration and mobility. This is an unusual phenomenon because the carrier concentration and mobility are generally inversely related^45–47^. The increase in the carrier concentration of the nanocomposite may originate from the high carrier concentration of the SWCNTs. The increase in mobility may occur due to the combined effects of the high mobility of the SWCNTs, an increase in the alignment of the Bi_2_Te_3_ nanoplates, and the integration between the Bi_2_Te_3_ nanoplates and SWCNTs. A similar phenomenon was observed in copper phthalocyanine/SWCNT hybrids^48^.

**Figure 6:**
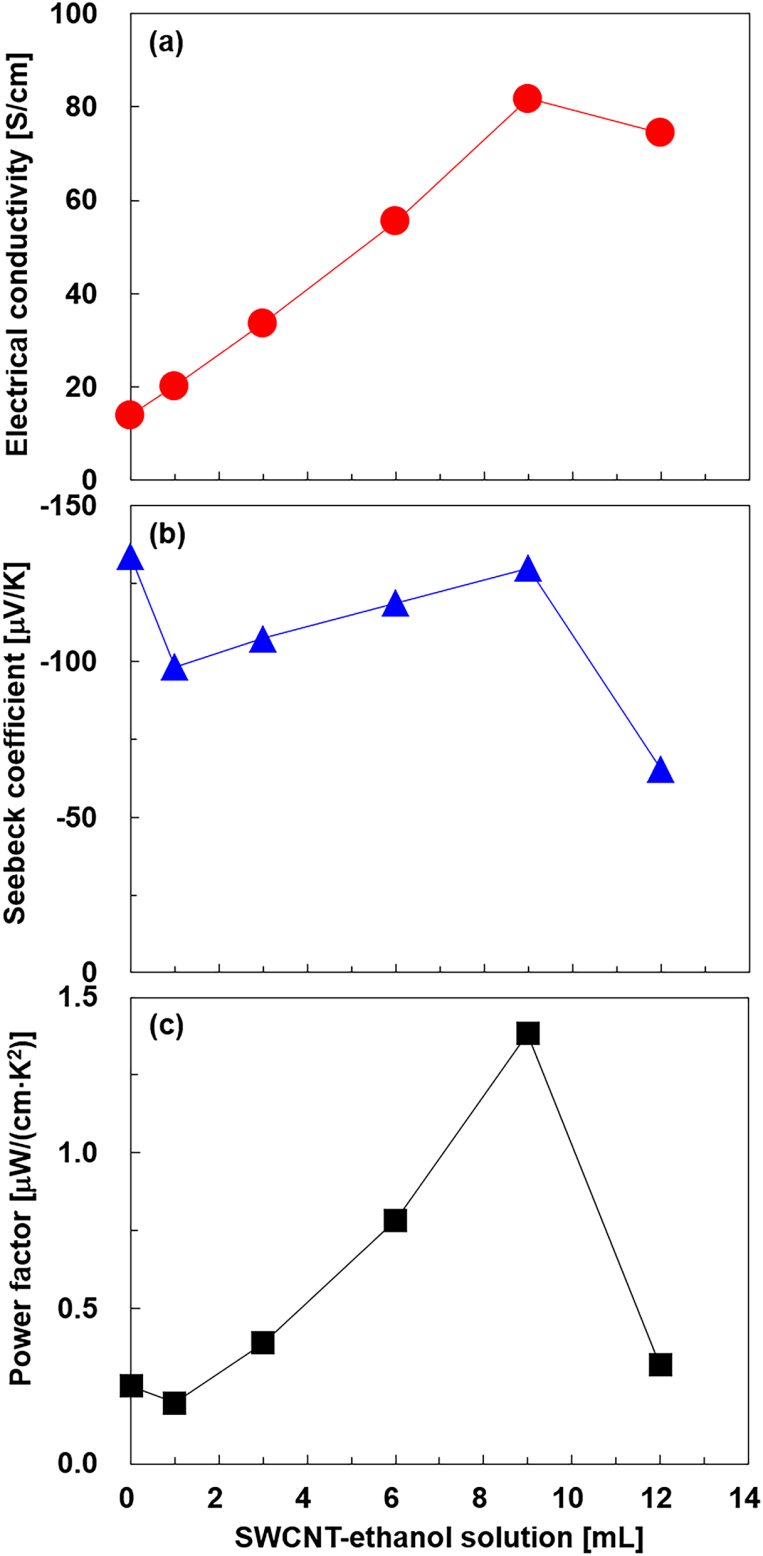
Thermoelectric properties of the nanocomposite films as a function of the SWCNT-ethanol solution: (**a**) electrical conductivity, (**b**) Seebeck coefficient, and (**c**) power factor.

Figure 6 shows the in-plane thermoelectric properties of the integrated nanocomposite films with varying SWCNT-ethanol solutions. As shown in Fig. 6a, the electrical conductivity of the films increased as the SWCNT-ethanol solution increased to 9 mL, owing to the increases in the carrier concentration and mobility. The electrical conductivity at a SWCNT-ethanol solution of 12 mL decreased compared to that at 9 mL, possibly because the sample at 12 mL resulted in a very fragile structure with microcracks that interrupted electronic transport. In Fig. 6b, the Seebeck coefficient of the film with no SWCNTs was –133.8 μV/K. With the addition of 1 mL of the SWCNT-ethanol solution, the Seebeck coefficient became less negative at – 98.3 μV/K because the SWCNTs in this study inherently exhibited a positive Seebeck coefficient^25,49^. When the SWCNT-ethanol solution increased from 1 to 9 mL, the Seebeck coefficient became more negative from − 98.3 to – 130.0 μV/K. This is a remarkable phenomenon considering that the added SWCNTs possess a positive Seebeck coefficient. In our previous study, in which SWCNTs were added to the films during drop-casting, but excluding solvothermal synthesis, the Seebeck coefficient became less negative as the SWCNT-ethanol solution increased^37^. The mechanism of this phenomenon is not clear, but one possible explanation is that the contact resistance between the Bi_2_Te_3_ nanoplates and SWCNTs decreased owing to the integration of the materials. This decrease in the contact resistance could contribute to reducing the voltage drop when the Seebeck voltage was generated in the film. This indicates that the Seebeck coefficient can be maintained at a relatively high value under conditions of low contact resistance. Similar phenomena were observed in thin-film thermoelectric generators^19,50^. The drop in the Seebeck coefficient at 12 mL was likely due to sample fragility. In Fig. 6c, the highest power factor of 1.38 μW/(cm K^2^) was achieved for the 9 mL SWCNT-ethanol solution because of its high electrical conductivity and high negative Seebeck coefficient. This value was 5.5 times higher than that of the film without SWCNTs.

In Table 1, we compare the thermoelectric properties of the integrated nanocomposite film with the maximum power factor in this study (SWCNT-ethanol solution of 9 mL) to those of the reference sample, which was a simple nanocomposite film formed in our previous study by adding SWCNTs during drop-casting, but excluding solvothermal synthesis^37^. A reference sample containing the same carbon mass concentration was chosen for comparison. Even though the carbon mass concentration was nearly the same in both samples, the integrated nanocomposite film in the present study exhibited a higher electrical conductivity and Seebeck coefficient. As a result, the power factor of the integrated nanocomposite film was 23 times higher than that of the simple nanocomposite film. Thus, we have demonstrated the effectiveness of the fabrication process for integrated nanocomposite films in which SWCNTs were added during the solvothermal synthesis.

**Table 1:** Comparison of thermoelectric properties of nanoplates/SWCNTs films.

Sample	Carbon mass conc. (wt%)	*σ* (S/cm)	*S* (µV/K)	*PF* (µW/(cm K^2^))	References
Integrated nanocomposite film adding SWCNTs during solvothermal synthesis (SWCNT-ethanol solution: 9 mL)	12.8	81.8	− 130.0	1.38	This study
Nanocomposite film adding SWCNTs during drop-cast	12.7	16.2	− 58.6	0.06	^37^

## Discussion

**Figure 7:**
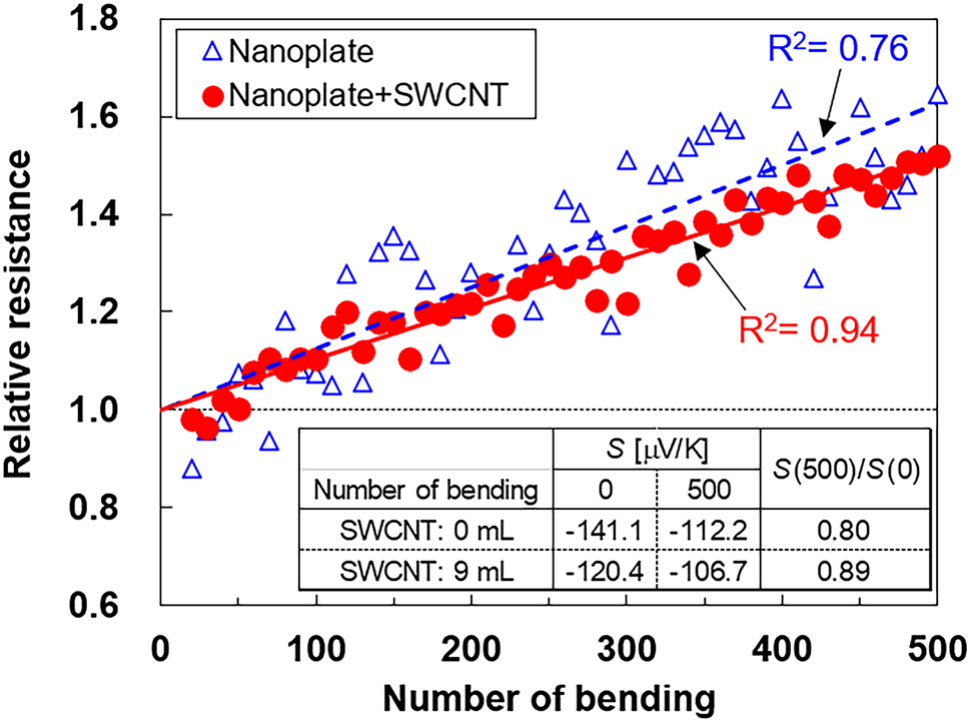
Relative resistance of the integrated nanocomposite film and the nanoplate film as a function of the number of bends. The Seebeck coefficients of these films at initial (no bends) and final (500 bends) conditions are shown in the insets.

To use integrated nanocomposite films practically, it is necessary to investigate the change in the thermoelectric properties under repetitive bending conditions^51–54^. Therefore, we performed bending tests by applying stress on the film with the highest power factor (SWCNT-ethanol solution of 9 mL) and on a nanoplate film with no SWCNTs as a reference. The images of the bending tests are shown in Supplementary Fig. S3. The relative resistance and Seebeck coefficient were measured as the films were repeatedly bent 500 times, as shown in Fig. 7. The resistance of the integrated nanocomposite film after the last bend was approximately 1.5 times higher than that of the film prior to bending; the resistance of the nanoplate film with no SWCNTs after bending was approximately 1.6 times higher than that of the film prior to bending. The relative resistance of the integrated nanocomposite film after bending appeared lower than that of the nanoplate film without SWCNTs. However, as the accuracy of the fitting line of the nanoplate film with no SWCNTs (R^2^ = 0.76) was low compared to the integrated nanocomposite film (R^2^ = 0.94), comparing the relative resistance of the two films is unreliable; thus, further studies are required. The variations in the plot of the integrated nanocomposite film were smaller than those of the nanoplate film with no SWCNTs. This phenomenon can be explained by the difference in the uniformity of the films. The integrated nanocomposite film exhibited a relatively high uniformity owing to the network of SWCNTs. In contrast, since the nanoplate thin film did not contain the SWCNTs, the network between the nanoplates was inadequate, and the current path lines altered when the measurement locations were changed from the bending test. We believe that this is the cause of the variation in the resistance value of the nanoplate thin film.

The inset of Fig. 7 presents the Seebeck coefficients of the integrated nanocomposite film and the nanoplate film with no SWCNTs at the initial (no bends) and final (500 bends) conditions. The Seebeck coefficient of the nanoplate film with no SWCNTs decreased by 20% between the initial and final conditions, while that of the integrated nanocomposite film decreased by only 11%. Therefore, we concluded that the deterioration of the thermoelectric properties by bending could be suppressed by adding SWCNTs in the films.

**Figure 8:**
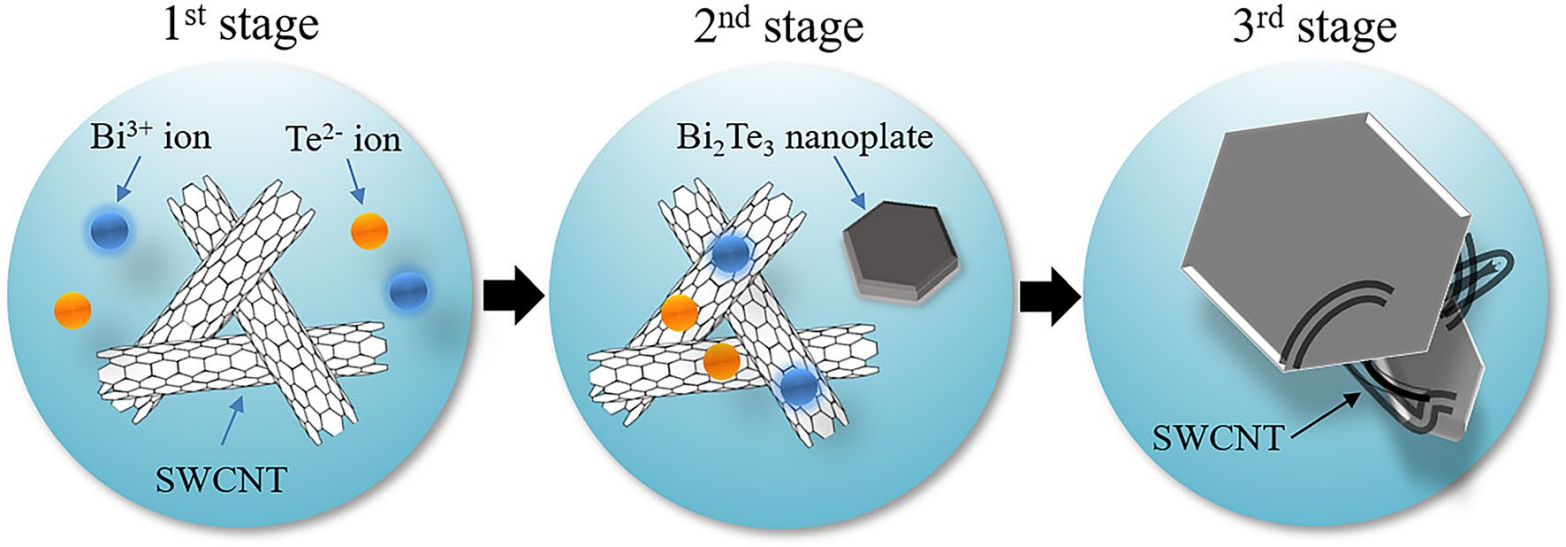
Schematic diagram of the growth mechanism of integrated nanocomposites composed of Bi_2_Te_3_ nanoplates and SWCNTs.

Here, we consider the growth mechanism of the integrated nanocomposites composed of Bi_2_Te_3_ nanoplates and SWCNTs, as shown in Fig. 8. In the first stage, Bi^3+^ ions, Te^2−^ ions, and SWCNTs separately exist in the precursor solution. In the second stage, some Bi^3+^ and Te^2−^ ions combine to form Bi_2_Te_3_ nanoplates, while others adhere to the surface of the SWCNTs. In the third stage, during the growth of the Bi_2_Te_3_ nanoplates, the SWCNTs were integrated with the nanoplates to form the nanocomposite. In addition, Bi_2_Te_3_ nanoplates were grown on the surface of the SWCNTs. Because of this growth mechanism, the nanoplates were tightly connected to the SWCNT_S_, leading to an increase in the electrical conductivity, Seebeck coefficient, and bending stability.

In summary, we fabricated integrated nanocomposites through the addition of SWCNTs during the solvothermal synthesis of Bi_2_Te_3_ nanoplates. Flexible films were prepared by drop-casting followed by thermal annealing. The mass concentration of carbon increased as the SWCNT-ethanol solution increased, and the crystalline phase of Bi_2_Te_3_ was maintained for all SWCNT-ethanol solutions. According to the SEM observations, the SWCNTs were well-integrated by the Bi_2_Te_3_ nanoplates. Owing to this structure, the electrical conductivity and Seebeck coefficient significantly increased compared to those of nanoplate films without SWCNTs. We compared the thermoelectric properties of the integrated nanocomposite film in this study to those of the nanocomposite film with similar carbon mass concentrations formed by simple mixing, where the SWCNTs were added during drop-casting but excluding solvothermal synthesis. The integrated nanocomposite film in this study exhibited high electrical conductivity and an n-type Seebeck coefficient. As a result, the power factor of the integrated nanocomposite film was 23 times higher than that of the nanocomposite film formed by simple mixing. This phenomenon might occur because the contact resistance between the Bi_2_Te_3_ nanoplates and the SWCNTs decreased due to the integration of the materials. In addition, this integration contributed to an increase in the stability of the thermoelectric properties after repeated bending.

## Methods

### Synthesis of nanocomposite materials

Nanocomposites with SWCNTs and Bi_2_Te_3_ nanoplates were prepared via solvothermal synthesis. The outline of this process is shown in Supplementary Fig. S4. The system consisted of a Teflon-lined stainless-steel autoclave and a hot plate with a magnetic stirrer. We used Bi_2_O_3_ (Fujifilm Wako Pure Chemical Co., > 99.9%), TeO_2_ (Kojundo Chemical Laboratory Co., Ltd., > 99.9%), ethylene glycol (Fujifilm Wako Pure Chemical Co., > 90.0%), polyvinyl pyrrolidone (PVP; Fujifilm Wako Pure Chemical Co., K30, M_s_ ~ 40,000), sodium hydroxide (NaOH; Fujifilm Wako Pure Chemical Co., > 97.0%), and SWCNTs (Zeon Co., ZEONANO SG101) to prepare the precursor liquid solution. The procedure for the synthesis of the nanocomposites was as follows: 0.4 g of PVP was dissolved in 18 mL of ethylene glycol, followed by the addition of Bi_2_O_3_ (20 mM), TeO_2_ (70 mM), 2 mL of aqueous NaOH solution (0.5 M), and 1–12 mL of SWCNT-ethanol solution (0.2 wt%). The resulting precursor solution was sealed in an autoclave. The autoclave was then heated to 200 °C and maintained at that temperature for 4 h, while the solution was stirred at 500 rpm. After synthesis, the products were cooled naturally to below 50 °C. The products were collected by centrifugation and washed several times with distilled water and pure ethanol. Finally, they were dried under vacuum at 60 °C for 24 h.

### Preparation of nanocomposite films

Nanocomposite films were prepared by drop-casting followed by thermal annealing. The basic procedure is described in our previous reports^55,56^. The prepared nanocomposites (30 mg) were ultrasonically mixed with 3 mL of methanol. The solution was then drop-casted on a polyimide substrate in an aluminum enclosure (22 × 12 mm^2^ area, 20 mm wall thickness) to flatten the solution at the center of the substrate. After drying in air, the nanocomposite thin films were thermally annealed at 250 °C for 1 h in an electric furnace. The electric furnace was filled with a mixture of argon (95%) and hydrogen (5%) at atmospheric pressure, and the gas-flow rate was maintained at 1.0 SLM throughout annealing. Following thermal annealing, the samples were naturally cooled to below 50 °C in the furnace.

### Material characterization

The atomic composition of the samples was estimated using EPMA (EPMA-1610, Shimadzu). The compositions of the samples were calibrated using the ZAF4 program supplied with the EPMA-1610 device. The microstructures of the samples were characterized by scanning electron microscopy (SEM, Hitachi S-4800) and transmission electron microscopy (TEM, JEOL JEM-2100F). The crystallographic characteristics of the samples were characterized by X-ray diffraction (XRD, Rigaku MiniFlex600).

### Thermoelectric performance and bending flexibility measurements

The carrier concentration (*n*) and mobility (*μ*) were measured near 300 K using the van der Pauw method (HM-055, Ecopia). The in-plane electrical conductivity (*σ*) of the samples was measured using a four-point probe method (Napson, RT-70V). The in-plane Seebeck coefficient (*S*) of the samples was measured near 300 K. One end of the thin film was connected to a heat sink, and the other end was connected to a heater. The in-plane Seebeck coefficient *S* of the thin films was measured at approximately 300 K using homemade equipment^50^. The measurement procedure and a schematic diagram of the measurement system are provided in the Supplemental information (Supplementary Fig. S5). The in-plane power factor (*σS*^2^) was obtained from the experimentally measured Seebeck coefficient and electrical conductivity. Bending tests were performed when the two samples (the integrated nanocomposite film and nanoplate film with no SWCNTs) were attached to the surface of the bending plastic with a radius of 20 mm. The basic procedure is described in our previous report^57^. When the samples were repeatedly bent 500 times, the resistance was measured every tenth time. In addition, the Seebeck coefficient was measured at the initial and final bending conditions. No peeling behavior was observed in either sample after the bending tests were completed.

## Supplementary information

Supplementary file 1.

## Data Availability

The data that support the findings of this study are available from the corresponding authors on reasonable request.
