# Supplementary material for: Flexible thermoelectric films formed using integrated nanocomposites with single-wall carbon nanotubes and Bi2Te3 nanoplates via solvothermal synthesis
Source: Sci Rep. 2020 Oct 12;10:17031. doi: 10.1038/s41598-020-73808-4 (PMC7550342; doi:10.1038/s41598-020-73808-4)
Supplement: Supplementary file 1 [file 41598_2020_73808_MOESM1_ESM.docx]

**Flexible thermoelectric films formed using integrated nanocomposites with single-wall carbon nanotubes and Bi_2_Te_3_ nanoplates via solvothermal synthesis**

Hayato Yabuki, Susumu Yonezawa, Rikuo Eguchi, Masayuki Takashiri*

*Department of Materials Science, Tokai University, Hiratsuka, Kanagawa 259-1292, Japan*

*E-mail: takashiri@tokai-u.jp

**Supplemental information**

Figure S1. Surface TEM images of the nanocomposite films. (a), (b), (c), (d), (e), and (f) correspond to SWCNT amounts in the precursor solution of 0, 1, 3, 6, 9, and 12 mL, respectively.

Figure S2. HRTEM images of the nanocomposite films. (a), (b), (c), (d), (e), and (f) correspond to SWCNT amounts in the precursor solution of 0, 1, 3, 6, 9, and 12 mL, respectively. The parts surrounded by the dotted lines are places where many absorbates exist on the nanoplate surfaces.

Figure S3. Images of bending tests forming convex shape with a curvature radius of 20 mm. (a) Integrated nanocomposite films with the highest power factor (amount of SWCNTs in the precursor solution of 9 mL), (b) nanoplate film with no SWCNTs.

Figure S4. Schematic diagram of nanocomposites prepared via solvothermal synthesis.


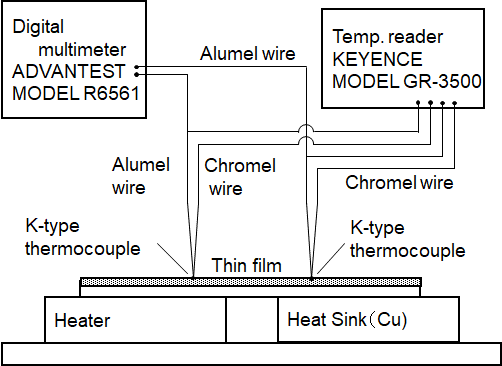


Figure S5. Schematic diagram of Seebeck coefficient measurement.

Two K-type thermocouples were used to measure the temperature difference (Δ*T*) and potential difference (Δ*V*) along the film. The measured Seebeck coefficient (*S_meas_*) was calculated as the ratio of Δ*V* to Δ*T*. However, *S_meas_* is the value including the Seebeck coefficient (*S* = -20.5 μV/K) of alumel wire (*S_wire_*) and that of the film (*S_film_*): *S_meas_* = *S_film_* + *S_wire_*. Therefore, to calculate only *S_film_*, the Seebeck coefficient of the alumel wire is subtracted from the *S_meas_*: *S_film_* = *S_meas_ − S_wire_*. We measured the *S_film_* using chromel wire (*S* = 20.5 μV/K) by the same procedure. Finally, we determined the Seebeck coefficient of the thin film from the average value of the Seebeck coefficients calculated using the alumel and chromel wires.
